# A recombinant rabies vaccine that prevents viral shedding in rabid common vampire bats (*Desmodus rotundus*)

**DOI:** 10.1371/journal.pntd.0010699

**Published:** 2022-08-26

**Authors:** Elsa M. Cárdenas-Canales, Andres Velasco-Villa, James A. Ellison, Panayampalli S. Satheshkumar, Jorge E. Osorio, Tonie E. Rocke

**Affiliations:** 1 Department of Pathobiological Sciences, School of Veterinary Medicine, University of Wisconsin-Madison, Madison, Wisconsin, United States of America; 2 Poxvirus and Rabies Branch, Division of High-Consequence Pathogens and Pathology, National Center for Emerging and Zoonotic Infectious Diseases, Centers for Disease Control and Prevention, Atlanta, Georgia, United States of America; 3 US Geological Survey, National Wildlife Health Center, Madison, Wisconsin, United States of America; George Washington University School of Medicine and Health Sciences, UNITED STATES

## Abstract

Vampire bat transmitted rabies (VBR) is a continuing burden to public health and agricultural sectors in Latin America, despite decades-long efforts to control the disease by culling bat populations. Culling has been shown to disperse bats, leading to an increased spread of rabies. Thus, non-lethal strategies to control VBR, such as vaccination, are desired. Here, we evaluated the safety and efficacy of a viral-vectored recombinant mosaic glycoprotein rabies vaccine candidate (RCN-MoG) in vampire bats (*Desmodus rotundus*) of unknown history of rabies exposure captured in México and transported to the United States. Vaccination with RCN-MoG was demonstrated to be safe, even in pregnant females, as no evidence of lesions or adverse effects were observed. We detected rabies neutralizing antibodies in 28% (8/29) of seronegative bats post-vaccination. Survival proportions of adult bats after rabies virus (RABV) challenge ranged from 55–100% and were not significantly different among treatments, pre- or post-vaccination serostatus, and route of vaccination, while eight pups (1–2.5 months of age) used as naïve controls all succumbed to challenge (P<0.0001). Importantly, we found that vaccination with RCN-MoG appeared to block viral shedding, even when infection proved lethal. Using real-time PCR, we did not detect RABV nucleic acid in the saliva samples of 9/10 vaccinated bats that succumbed to rabies after challenge (one was inconclusive). In contrast, RABV nucleic acid was detected in saliva samples from 71% of unvaccinated bats (10/14 sampled, plus one inconclusive) that died of the disease, including pups. Low seroconversion rates post-vaccination and high survival of non-vaccinated bats, perhaps due to earlier natural exposure, limited our conclusions regarding vaccine efficacy. However, our findings suggest a potential transmission-blocking effect of vaccination with RCN-MoG that could provide a promising strategy for controlling VBR in Latin America beyond longstanding culling programs.

## Author summary

Rabies is a fatal, zoonotic disease of global importance. In Latin America, the common vampire bat is responsible for most of the rabies outbreaks in livestock, and for decades, culling vampire bats has been an approach to control rabies. However, culling is not a sustainable practice, and non-lethal control strategies, such as the use of oral rabies vaccines, offer an alternative to manage the disease at the reservoir level. Our vaccine candidate, RCN-MoG, uses raccoon pox (RCN) as a viral vector for a mosaic gene expressing rabies glycoprotein (MoG). We orally or topically vaccinated wild-caught vampire bats with RCN-MoG and found that only a small proportion produced neutralizing antibodies after vaccination, and their survival to challenge with rabies virus (a measurement for vaccine protection) was not significantly improved compared to unvaccinated bats. Notably, however, vaccination with RCN-MoG had a blocking effect against viral shedding in the saliva of infected bats that succumbed to rabies. Our findings revealed a previously unknown but important benefit of vaccinating vampire bats: preventing viral shedding by rabid bats, which could reduce the spread of disease among bats and other animals. These observations should encourage further research, especially under field conditions.

## Introduction

Few diseases have haunted humanity as rabies has done for millennia [1,2]. Rabies is a fatal zoonotic disease caused by the rabies virus (RABV), an RNA-negative strand virus of the Lyssavirus family, capable of infecting a wide range of mammalian hosts [3]. Since early studies on rabies, numerous scientific advances have contributed to controlling and preventing this disease [1]; most prominent are the development of injectable rabies vaccines for humans and domestic animals. To reduce rabies in wild animal reservoirs, oral recombinant vaccines delivered via baits have been effective in wild carnivores in North America and Europe [4,5]. Yet, RABV is maintained in numerous other reservoir species worldwide (e.g., bats) [6], contributing to the complexity of its epidemiology and control.

In Latin America, the common vampire bat (*Desmodus rotundus*) is currently the main reservoir of RABV [7]. Unique to this region of the continent, *D*. *rotundus* feeds solely on blood (via bite), a behavior that creates an ideal route of transmission for RABV. Thousands of livestock animals and humans are bitten by vampire bats and potentially exposed to RABV every year [8], causing a substantial burden on public health and animal production sectors [6,7,9,10]. Culling vampire bat populations is a strategy widely used by Latin American countries to control vampire bat transmitted rabies (VBR), but several studies have concluded that this practice is not effective or sustainable [11,12], as evidenced by the expansion of rabies into new areas [11,13]. Moreover, beneficial bats are sometimes culled indiscriminately during these operations.

Motivated by the increasing need to identify novel control strategies, this study evaluated the effect of oro-topical vaccination of vampire bats against RABV using a recombinant raccoon poxvirus (RCN) expressing a mosaic gene of the rabies glycoprotein G (MoG). This vaccine candidate (RCN-MoG) was developed and tested previously in captive big brown bats (*Eptesicus fuscus*) [14,15] and shown to be safe and effective in protecting them against rabies via both oral and topical routes of administration, prerequisites for feasible application to wild bats [4]. While the concept of vaccinating vampire bats, potentially improving their survival and increasing their abundance, is controversial, it has been explored before using vaccinia rabies glycoprotein vaccines developed for terrestrial carnivores [16–19] but never advanced to field testing. The use of vaccinia as a vaccine vector has raised safety concerns for both humans [20] and livestock [21], the preferred prey of vampire bats. Although similar to vaccinia, RCN is a safer viral vector, shown to be more attenuated or avirulent in laboratory animal models while still highly immunogenic [22].

We conducted rabies vaccination and challenge studies using common vampire bats captured in México and transported to a biosafety level-3 animal facility in the United States. Our objectives were to assess the overall safety of RCN-MoG for vampire bats delivered orally or topically, humoral immunity elicited by vaccination, protection against RABV challenge, and viral shedding by rabid bats. We were particularly interested in the potential blocking effect of vaccination on RABV shedding, as documented previously in vampire bats vaccinated after exposure to RABV [23], and whether bats seropositive for rabies virus neutralizing activity (RVNA) (and thus likely exposed previously) survived RABV challenge at higher rates than seronegative bats.

Having a large cohort of wild-caught vampire bats from a diverse population available for vaccination studies is a unique opportunity. For this reason, we tried to leverage the study to address several additional questions beyond vaccine efficacy using specific cohorts of our captive population (e.g., vaccine safety during pregnancy, transfer of topically applied vaccine among female cage mates, and antibody decline after RABV exposure), which required different experimental parameters and were conducted over different timelines. We encountered challenges during the study, and it was difficult to control all intrinsic factors, as expected when working with wild animals of unknown disease exposure history. Thus, we present results within broad categories (e.g., grouped by treatment received) and indicate relevant information more specifically for some individuals or groups.

## Methods

### Ethics statement

The Mexican Secretariat of Environment and Natural Resources (SEMARNAT) approved the capture and export of bats under permit numbers SGPA/DGVS/003242/18 and 44333, and we imported the bats under Centers for Disease Control and Prevention import permit number 2018-04-108. The Institutional Animal Care and Use Committee at the U.S. Geological Survey National Wildlife Health Center (NWHC), Madison, WI, USA, approved all husbandry and experimental procedures (protocol number EP180418).

### Vampire bats

The common vampire bats used in this study were wild-caught and had an unknown history of exposure to RABV during their lifetime. Bats were captured in San Luis Potosí, México, in July–August 2018 using mist nets placed at cave entrances and known roosting areas after sunset. We captured 93 vampire bats (of mixed sex and age) during a six-week field period and transported them to NWHC, where the experiment took place in early September. The bats were transported in 10 custom-made containers (8–14 bats per container depending on their source, with males and females separate) (see [24] for details on transport). One male bat (#563) died in transit and was confirmed positive for RABV by direct fluorescent antibody testing (DFA) [23]; another (#565) in the same shipping container died from rabies 18 days later. Up to 10 other male vampire bats transported or housed with these 2 bats may have been exposed to RABV. Two females (#573 and #641) also died after arrival (confirmed positive for RABV by DFA); up to 8 other female bats may have been exposed due to contact with these bats. One other male died from causes not related to rabies (confirmed negative for RABV by DFA), leaving 88 bats.

We housed the bats in a biosafety level-3 animal facility for the duration of the study (September 2018–June 2019). Males and females were housed separately in mesh cages described previously [23,24] to avoid reproduction, with males in three mesh cages and females in two. Bats were grouped according to their original capture location. We followed recommended husbandry guidelines for vampire bats [25], feeding them a diet of citrated cow blood and offering water *ad lib*. Bats were housed under controlled temperature (28–34°C) and humidity (>40%). Eight females were captured at an early stage of pregnancy, and each gave birth, adding eight animals to the study. The initial acclimation and quarantine period lasted approximately 100 days before the start of the vaccine study (Dec 2018), an adequate duration given the long rabies incubation periods known in bats [26–28]. During this time, health checks were performed weekly. On days 34–57 after arrival, we collected a blood sample from all bats from the cephalic vein as previously described [29] (i.e., baseline) for serologic assays.

### Detection of rabies virus neutralizing antibodies (RVNA) and assessment of previous RABV exposure

RVNA was used as an indicator of previous RABV exposure. To detect RVNA, we conducted the *in vitro* cell-based functional neutralizing assay Rapid Fluorescent Focus Inhibition Test (RFFIT) [30]. Due to the small volume of blood that can be obtained from bats (up to 200 μL), we performed a modified micro-RFFIT using plasma or serum samples as described previously [31]. The test results are expressed in end-point reciprocal titers by the Reed-Muench method [32] and converted to International Units per milliliter (IU/mL) by comparison to a reference standard rabies immune globulin (SRIG) [30]. Given the lack of a defined cut-off value in bats [33], in this study, we assigned a sample “positive” when virus neutralization was evident in >50% of the fields at a 1:10 dilution of the test sample (equivalent to a ≥1:13 reciprocal end titer or >0.06 IU/mL). Blood samples collected at the baseline timepoint were screened for RVNA to determine which animals may have been previously exposed to RABV. Once all samples from individual bats were available at the end of the study, they were tested in the same micro-RFFIT run to allow comparisons over time, without inter-test variation, and those were the values analyzed.

### Viral vector recombinant mosaic glycoprotein vaccine candidate, RCN-MoG

Our vaccine candidate, RCN-MoG, and its cultivation were described previously [15]. Briefly, MoG was designed *in silico* from a selection of 664 available glycoprotein sequences from the RABV Phylogroup I that represent RABV variants most likely occurring in bats. Thus, a broad antigenic coverage was obtained. As a negative control, we used RCN expressing a luciferase gene (*luc*). Stocks for both RCN-MoG and RCN-*luc* were produced in Vero cells (ATCC #CCL-18) and quantified by plaque assays [14,15].

### Vaccine treatments

To evaluate the immune response elicited by oro-topical vaccination, we first separated the bats based on their baseline serostatus (Table 1). Using the micro-RFFIT, we detected evidence of RVNA in 13 of 88 available adult bats (12 males and 1 female) at the baseline timepoint (1–2 months post-arrival) and designated these bats as seropositive-at-baseline, likely exposed to RABV in the past. The rest were designated as seronegative-at-baseline. The bats were assigned to their experimental groups based on this designation and then randomly allocated into subgroups according to treatment to be administered (e.g., vaccine-RCN-MoG, control-RCN-*luc*, no treatment) and vaccination route (direct, by instillation in the oral cavity, or indirect, in a topical vehicle). Males and females were treated separately.

**Table 1:** Experimental setup of common vampire bats grouped by sex, rabies virus neutralizing antibodies serostatus at baseline time point, treatment, and route. Males received 1x10^8^ PFU/mL orally of RCN-MoG (vaccine) or RCN-*luc* (control), or 2x10^8^ PFU/mL of RCN-MoG mixed in 0.5 mL of glycerin jelly for topical vaccine. Topically vaccinated females received 5x10^8^ PFU/mL RCN-MoG in 1 mL^a^. Eight captive-born pups did not receive treatment but were included in the RABV challenge. For this group F = female and M = male, one pup was not sexed. The sampling schedule differed for male and female and seropositive groups, as indicated in the last column.

Sex	Baseline serostatus	Treatment	Route	Initially included (n)^b^	Challenged (n)^c^	Sampling schedule (days post-vaccination)
Male	Seronegative	RCN-MoG	oral	21(8 ^d^)	17	Blood: 21, 71, 113-114, terminal sample^i^ Oral swabs: every 3 days, daily if clinical signs observed, at death
			topical	14 (9 ^d^)	14	
		RCN-*luc*	oral	12	12	
	Seropositive	RCN-MoG	oral	6	6	Blood: 28, 112, terminal sample Oral swabs: weekly, daily if clinical signs observed, at death
			topical	1^e^	1	
		RCN-*luc*	oral	5	4	
			topical	0	1 ^f^	
Female	Seronegative	RCN-MoG	topical	6	6	Blood: 30, at 33 days post-challenge, terminal sample Oral swabs: Weekly, daily if clinical signs observed, at death
		Transfer^g^	in-contact	8	8	
		No treatment^h^	-	8	8	
Pups (2M/5F)	Seronegative	No treatment	-	8	8	Blood: before RABV challenge (considered baseline) and terminal sample) Oral swabs: same as females

^a^ Vaccine dose is measured in plaque forming units per mL (PFU/mL) of the raccoon pox viral vector.

^b^ “n” is the initial number of bats that received treatment. This number changed later due to the death of subjects and the change in serostatus after the natural rabies outbreak occurring in December 2018. These bats are included in the serology analysis.

^c^ Bats were challenged in April 2019 with 10^3.3^ median tissue culture infective dose (TCID_50_/mL) of a heterologous (coyote) RABV strain.

^d^ A subset of bats from the oral and topically vaccinated groups (8 and 9 bats, respectively) were boosted 100 days after initial vaccination, using the same dose and route as previously administered

^e^ This topically vaccinated male bat was initially seronegative at baseline and grouped accordingly, but on retesting several times, it was found to be seropositive at baseline; therefore, it was reclassified.

^f^ This bat (#582) was initially seronegative at baseline and received RCN-*luc* topically but seroconverted after exposure during the outbreak; thus, considered seropositive for the survival analysis

^g^ Eight females were housed with four other females that received RCN-MoG topically to measure the transfer of vaccine by contact.

^h^ One of the females in this group was the only seropositive at the baseline time point; it was included in this group.

^i^ Terminal samples were those collected at the date of death or the end of the study.

Male bats, seronegative at baseline, were vaccinated orally or topically or received a placebo. For oral delivery of vaccine, 1x10^8^ plaque forming units (PFU) of RCN-MoG vaccine or RCN-*luc* (placebo), diluted in PBS, was dropped slowly into the mouth in 100 μL volumes by pipette. For topical delivery in males, 2x10^8^ PFU of RCN-MoG or RCN-*luc* was mixed into 0.5 mL of laboratory-grade glycerin jelly (Carolina Biological Supply, Burlington, North Carolina, USA) that included Rhodamine B (RB), a biomarker commonly used in wildlife [34], at a concentration of 0.06%. The doses selected were based on previous work with the same vaccine in big brown bats [15]. We applied the mixture onto the chest and back of the bats using a needleless syringe containing a single dose, prepared in advance, to allow consumption during grooming. We collected hair 7 days post-vaccination (dpv) and examined it under fluorescent microscopy (Nikon SMZ1270, at excitation and emission wavelengths of 540 and 625 nm, respectively) to detect the presence of metabolized-RB, observed as fluorescence in the hair follicle. We assumed vaccine uptake if the hair sample was positive for RB. A subset of seronegative males (8 oral and 9 topically vaccinated) was boosted 100 dpv, using the same dose and route to determine if boosting would increase RVNA titers and enhance protection against challenge.

Male bats seropositive at baseline were vaccinated orally or received a placebo (RCN-*luc*) as described above and were used to identify the potential effect of vaccination in previously exposed bats (e.g., boost effect). One of these bats (#628, seropositive-at-baseline, control) died before RABV challenge of causes not related to rabies as confirmed by negative DFA in brain tissue; we only report serology results at baseline and 28 dpv for this bat.

We used the female group to assess vaccine safety upon topical application during pregnancy and lactation and potential vaccination due to the transfer of vaccine-laden jelly between bats. Four females received 5x10^8^ PFU of RCN-MoG (2.5 x the dose applied to the males) in 1 mL of glycerin jelly-RB mixture at a 0.09% concentration; they were co-housed with eight untreated females (Table 1) to investigate the possibility of transfer of vaccine. Hair was collected periodically from all individuals (as early as 3 days after application) to assess uptake of RB, as described above. Untreated females were considered “in-contact,” and only considered vaccinated if they were RB positive. Due to veterinary issues unrelated to the experimental protocols (chronic inflammation of an eye that required prolonged treatment), two other females were topically vaccinated but remained isolated for most of the study.

To assess the safety of RCN-MoG in vampire bats, we observed bats daily after vaccination for signs of clinical disease or lesions (e.g., skin lesions) potentially caused by the viral vector, and in females, for effects on gestation and survival of their pups. We periodically collected blood samples for serology after vaccination at 21–28, 30, 71, and 112–114 dpv (all pre-challenge). A post-challenge (terminal) sample was collected from surviving bats at the end of the study or at the time of death for those succumbing to rabies. An additional blood sample was collected from 15 females 33 days post RABV challenge (dpc). The large number of bats in the study necessitated sampling sessions over several days.

### Experimental rabies challenge

For the challenge, we administered a heterologous RABV variant (10^3.3^ median tissue culture infective dose-TCID_50_/mL) in a volume of 100 μL, injected intramuscularly into each masseter muscle (50 μL on each side) while the bats were sedated with isoflurane. The challenge RABV (cRABV) selected was a coyote variant isolated from a naturally infected coyote in Texas and was provided by the CDC under a collaborative research agreement (D-615-15). The use of a heterologous RABV variant allowed us to distinguish mortality due to the challenge virus from naturally occurring VBR infections at the time of challenge.

To avoid cage allocation bias, we reassigned bats of the same sex into new cages before the challenge, so individuals from different treatments would be mixed, and we gave them a week to adapt. The smaller group of female bats (n = 22) was challenged first in February 2019 (31 dpv) to confirm the challenge dose was adequate. One pup was challenged with this cohort, as it was volant and one month old at the time. Males (n = 55) were challenged in April 2019 (112 or 127 dpv, depending on when they received the vaccine). The rest of the pups (n = 7; all unvaccinated) were challenged at the same time (at an estimated age range of 2 to 2.5 months) and were included to confirm the lethality of the challenge virus. After challenge, we observed bats for clinical signs of infection/mortality twice daily. Bats showing clinical signs of self-mutilation, respiratory distress, ataxia, paralysis, or weight loss of more than 20% of their mass were euthanized. Surviving bats were euthanized at 49–50 dpc, necropsied, and tested for RABV. Surviving females with pups were kept alive longer (up to 99 days) and were euthanized when their pups succumbed to rabies.

### Rabies confirmation by Direct Fluorescent Antibody Test (DFA) and molecular diagnostic techniques

We performed the direct fluorescent antibody test (DFA) for RABV diagnosis in brain impression smears from all bats, according to methods described elsewhere [30,35], to confirm death by rabies. Briefly, slides were fixed in acetone at -20° C, and a rabies fluorescein isothiocyanate (FITC)-labeled monoclonal antibody (Fujirebio Diagnostics, Malvern, Pennsylvania, USA) was added to each well and incubated at 37° C under 5% CO_2_ for 30 minutes. Slides were washed with PBS and observed under fluorescent microscopy. We used a second conjugate (Light Diagnostics Rabies DFA Reagent 5200, Millipore, Burlington, Massachusetts, USA) as a negative control to discriminate for background fluorescence. To confirm mortality was due to cRABV of canine origin (coyote), we used end-point PCR and Sanger sequencing (S1 Fig) on brain tissue from eight DFA positive bats.

### Detection of RABV in saliva and salivary glands

We conducted a real-time RT-PCR LN34 pan-Lyssavirus assay as described elsewhere [36,37] using the published cut-off value, to test for the presence of RABV nucleic acid in salivary glands and saliva (oral swabs) from bats that died or presented signs of rabies after challenge. We also tested the salivary glands of five bats that survived the challenge and were DFA negative as negative controls. The saliva sample was obtained by swabbing the oral cavity with sterile polyester tipped applicators (Puritan, Guilford, Maryland, USA). Modifications to the RT-PCR protocol included using 8.5 μL of RNA per reaction and an iCycler instrument (BioRad, Hercules, California, USA). Cycle threshold (Ct) values ≤ 35 were considered positive. Values 35–40 were considered inconclusive, indicating low viral load, insufficient sample, or possible RABV-RNA cross-contamination as indicated by the published guidelines [36]. RNA extraction was performed using Direct-zol RNA Miniprep kit (Zymo Research, Irvine, California, USA) following the manufacturer´s instructions.

We attempted virus isolation in cell culture from RT-PCR positive samples to detect infectious RABV. We followed standard methods for the rabies tissue culture infection test (RTCIT) [30]. Given the limited amount of tissue available, we used undiluted tissue homogenates to infect BHK-21 cells treated with DEAE-Dextran (Millipore-Sigma, St. Louis, Missouri, USA) at a concentration of 10mg/ml [38]. The cell-virus suspension was cultured in 25cm^2^ flasks (Corning, Corning, New York, USA) at a density of 2.4x10^6^ cells/mL and incubated at 37° C under 5% CO_2_ for 48 hours. Along with the flasks, slides were prepared for each sample to monitor cell infection at 48 and 72 hours. Cell infection was determined by DFA as described above and considered negative if no fluorescence was detected after three cell passages performed every 72 hours.

### Statistical analysis

To analyze RABV neutralizing activity response due to vaccination and to compare differences between treatment groups from the serially sampled seronegative and seropositive bats, we fit a linear mixed-effects model, treating bats as the random effect, using the R-package “lme4” in R (R Core Team, 2020). Males and females were analyzed separately. We checked residuals for ANOVA assumptions and used a log_10_ transformation of the RVNA titers (IU/mL) to ensure model assumptions were met.

We used Kaplan-Meier analysis in GraphPad Prism version 9.0 for Windows (GraphPad Software, San Diego, California, USA) to analyze differences in survival of bats by treatment (males, classified by baseline serostatus initially, and females separately). Given the small sample size of the remaining subgroups (i.e., males orally or topically vaccinated or boosted), we combined them into broader categories, such as “vaccinated”, if preliminary Kaplan-Meier analysis showed no differences in survival among these subgroups. We also used Kaplan-Meier analysis to compare differences in survival of all vampire bats, excluding the pups, based on overall serostatus prior to challenge (positive at any time point prior to challenge compared to those that remained negative throughout the experiment).

Fisher´s exact test was used to analyze viral shedding data from bats that succumbed to challenge (n = 27), with all bats included (males, females, and pups) and to determine the association of RABV shedding and the manifestation of clinical signs with treatment (both binomial variables). A *P* value of <0.05 was considered significant.

Data used to perform analyses for this study are available online at https://doi.org/10.5066/P9KNHW1P [39].

## Results

### Naturally occurring rabies outbreak in captive vampire bats

Between December 10, 2018 (~3 months after arrival at NWHC and before administering any vaccine to bats) and January 30, 2019 (after the vaccination experiment was already under course), we observed a natural rabies outbreak in a single cage of male bats that was unrelated to earlier rabies mortalities and demonstrated to have been caused by a different RABV lineage [23]. Details of this outbreak, including clinical presentation, vaccination timeline, RABV shedding and RABV typing, were published elsewhere [23], and some additional details are included here in Table 2. Seventeen bats were co-housed with the index case (#576) and potentially exposed to RABV shed in saliva of rabid bats (confirmed later). Signs of aggression were recorded among the group. Overall, 10 succumbed to infection, including bats that had been orally vaccinated with RCN-MoG as part of the planned experiment (Table 2). Of the seven surviving bats, four had been seropositive-at-baseline and 3 seronegative-at-baseline before the outbreak, and all but 1 had seroconverted by 52 days after the index case. Except for bat #582 (seronegative, received RCN-*luc* topically), the rest were vaccinated orally during the outbreak. These seven surviving bats were challenged with cRABV with the other male bats included in the challenge experiment approximately 3 months after the outbreak, and all survived (Table 2).

**Table 2:** Male vampire bats involved in the natural rabies outbreak, from Dec 10, 2018–Jan 30, 2019 (n = 17). All were housed together with the index case (bat #576). Bats were initially assigned to treatment groups based on the detection of RVNA at the baseline timepoint (obtained on Nov 2, 2018). A blood sample was obtained from most bats to determine RVNA titers approximately 25–30 days after the index bat died and in surviving bats 52 days after the index case. RVNA are expressed in IU/mL. Bats surviving the natural outbreak were challenged with cRABV on April 25, 2019, and only data from those bats are included in analyses for this study.

Bat ID	Treatment assigned	RVNA baseline	RVNA 25–30 days after exposure	RVNA 52 days after exposure	Date of death	cRABV challenge survival
576	Index bat	0.06	-	-	12/10/18	-
677	died before treating	0.06	-	-	12/30/18	-
605	Control	0.06	-	-	1/5/19	-
601	RCN-MoG	0.06	0.06	-	1/17/19	-
678	RCN-MoG	0.06	0.06	-	1/24/19	-
584	Control	0.06	0.06	-	1/26/19	-
610	Control	0.06	0.06	-	1/29/19	-
676	Control	0.06	0.06	-	1/29/19	-
679	RCN-MoG	0.06	-	-	1/29/19	-
604	RCN-MoG	0.06	3.40	-	1/30/19	-
582	Control	0.06	-	1.26	survived	survived
579	RCN-MoG	0.06	0.06	0.06	survived	survived
583	RCN-MoG	0.06	-	2137.00	survived	survived
580	RCN-MoG	0.07	12.23	2.00	survived	survived
575	RCN-MoG	0.17	27.30	85.50	survived	survived
581	RCN-MoG	0.08	3.42	14.00	survived	survived
585	RCN-MoG	0.08	0.16	0.69	survived	survived

### Safety of RCN-MoG for vampire bats

Ultimately, more than 50 bats received, or were exposed to, RNC-MoG as part of this study. Apart from a slight decrease in blood consumption on the day of vaccination, no significant changes were reported in husbandry or behavior. None of the bats showed any adverse effects to vaccination. Four pregnant females were vaccinated topically; all successfully weaned their pups later.

### Detection of RVNA after vaccination in experimental groups

**Fig 1:**
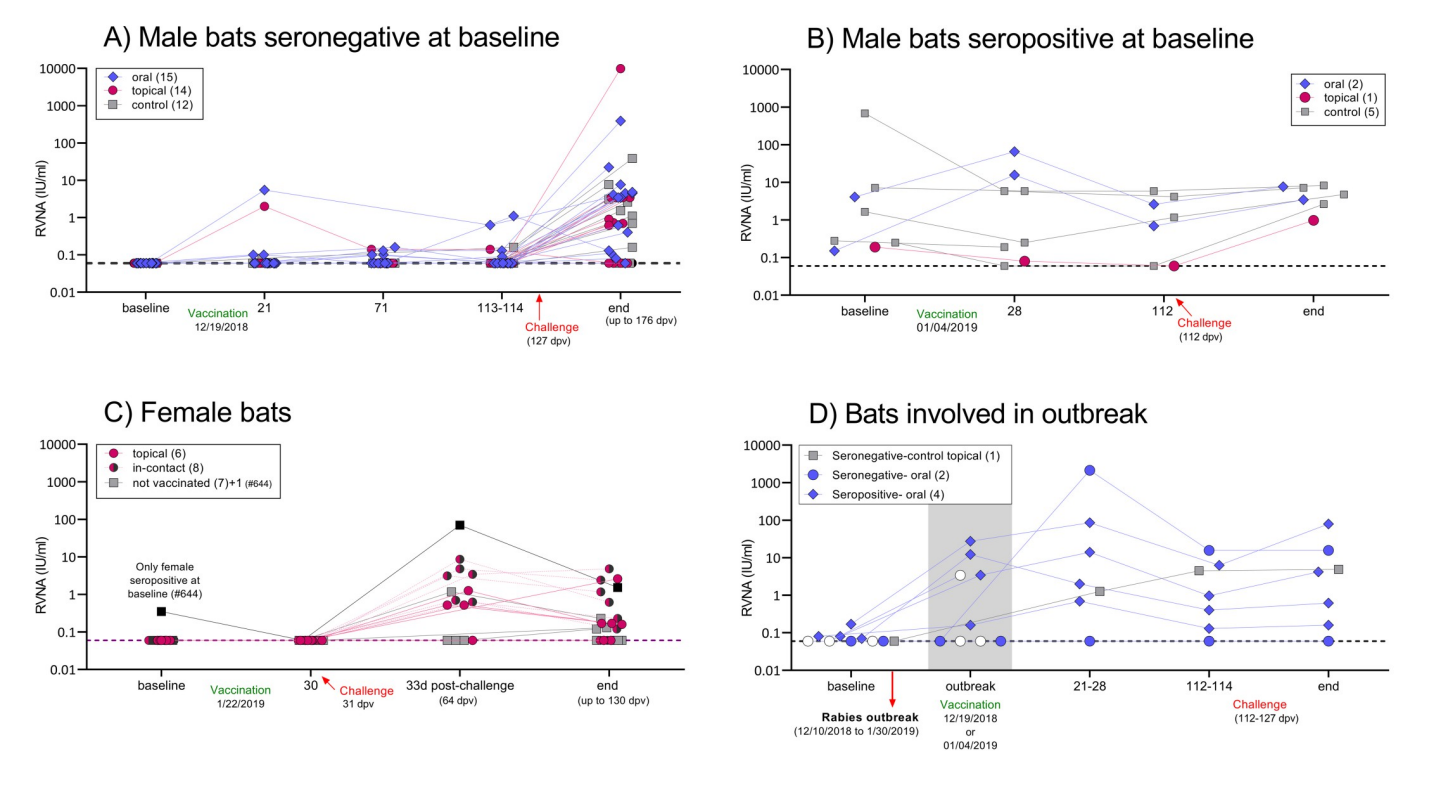
Detection of rabies virus neutralizing antibodies (RVNA) in individual vampire bats at different time points after vaccination and challenge with a heterologous (coyote) strain of RABV in A) male vampire bats seronegative at baseline, B) male vampire bats seropositive at baseline, C) females, showing an additional timepoint sample 33 days after challenge with cRABV, and D) bats that were involved in the natural rabies outbreak (RABV strain of vampire bat origin). In D, white-filled points represent three bats that died in the outbreak but were vaccinated and had a sample available for RVNA assessment. Events such as vaccination, the occurrence of a natural rabies outbreak within the captive colony, and the end of the study are indicated on the x-axis. Group size is indicated in parenthesis. The dotted line indicates the cut-off value of 0.06 UI/mL used in this study.

Seroconversion rates after vaccination were low and not consistent over time (Fig 1). Only 21% (6/29) of males seronegative at baseline developed RVNA at some point, and titers were generally low (Fig 1A). All topically vaccinated bats were considered positive for uptake, as indicated by the presence of RB in hair samples inspected, but only one seroconverted. After boosting, 2 bats (oral) developed RVNA, increasing the overall seroconversion rate to 28% (8/29). Unexpectedly, bat #613 (seronegative at baseline, receiving RCN-*luc*), showed evidence of RVNA at the 21 and 114 dpv timepoints. The rest of the control males remained without detectable RVNA throughout the study. We did not detect an effect of treatment (P = 0.2) or timepoint (P = 0.6), nor an interaction effect (P = 0.6), on RVNA titers (IU/mL) after vaccination among treatment groups, seronegative-at-baseline.

Seropositive male bats that received vaccine orally showed an increase in RVNA titers by 28 dpv, while the controls and the one topically vaccinated bat showed a decline at the same timepoint (Fig 1B). None of the vaccinated females had detectable RVNA titers (Fig 1C) after vaccination (30 dpv) despite detecting RB in the hair of all (6/6) of the topically vaccinated and 37% (3/8) of the in-contact bats, indicative of vaccine uptake. All pups were negative at baseline (obtained a few days before cRABV challenge) and terminal samplings. The occurrence of the natural outbreak in 17 male bats complicated our assessment of antibody responses in this group. All bats involved in the outbreak were excluded from their original groups for RVNA analysis and their outcome is shown in Fig 1D.

### Overall survival of vampire bats after challenge with heterologous cRABV

In total, we challenged 85 bats with cRABV: 77 adults, including the seven survivors of the natural outbreak, and eight captive-born pups. Of these, 27 bats (32%/ 19 adults and 8 pups), succumbed to rabies infection within the 49/50-day observation period, as confirmed by DFA of brain tissue samples. Molecular characterization of samples obtained from eight dead bats confirmed that cRABV of coyote origin was the variant that caused the mortality (S1 Fig).

The mean time from infection to death was 17.2 days (range: 9 to 50), including bat #598 (seronegative-at-baseline, topically vaccinated) that was alive at the end of the challenge observation period (50 dpc) but had started showing progressive clinical signs consistent with rabies ~ 3 weeks prior. It was confirmed RABV positive upon euthanasia, but since it may have survived longer, 50 days would be considered a minimum time-to-death. Mean time-to-death was the same between pups and adults (17.2 days), with one pup surviving to day 35 dpc. Clinical manifestation of rabies was present in both groups. For adults, the mean time-to-death was not significantly different (P = 0.07) between vaccinated and unvaccinated bats (20.9 and 13.1 days, respectively).

We observed clinical signs corresponding to both the furious and paralytic forms of rabies in our vampire bats, including self-mutilation, aggressive behavior, lack of grooming, incoordination, respiratory distress, and reluctance or inability to move. Most of the bats showing the furious form of rabies were not vaccinated (12/16, including 6 of the 7 pups that had clinical signs recorded) compared to 3/10 in the vaccinated group (Table 3), and this difference was significant (P = 0.043). All surviving bats showed no clinical signs of the disease and were confirmed DFA negative upon euthanasia.

**Table 3:** Detection of RABV in salivary gland and RABV shedding in saliva by LN34 RT-PCR in bats confirmed positive for rabies in brain tissue by the direct fluorescent antibody test. According to a rotating group schedule, saliva samples were obtained on day of death (0) if possible, daily if clinical signs were observed, or 1–3 days before death. Symbols are: + “positive”,–“negative”, and “inc” inconclusive. Inconclusive results are those with a Ct value from 35–40 and could indicate low virus load, insufficient sample, or possible cross-contamination. A Ct value with n/d is “not detected”, n/a means sample was not available. Age groups are J = juvenile, A = adult, and p = pup. Clinical signs are categorized as furious (F) or paralytic (P), and time to death is the number of days from RABV challenge until the bat died or was euthanized.

Bat ID	Sex	Age	Treatment	RT-PCR salivary gland		RT-PCR saliva (days before death)				RVNA terminal (IU/mL)	Clinical signs	Time to death (days)
				result	Ct value	0	-1	-2	-3			
**NON-VACCINATED**												
608	F	J	contact	–	n/d	+				0.06	F	11
696^a^	F	J	contact	+	27.08	n/a				0.06	F	11
893^a^	M	p	none	+	26.48	n/a				0.06	F	12
561^e^	M	A	control	+	29.12	–			inc	1.53	P	15
617	M	A	control	+	32.76	–			inc	0.06	P	14
891		p	none	+	32.06	–				0.06	F	13
894	F	p	none	+	32.62	–				0.06	F	35
692 ^a^	F	A	none	+	31.19	inc				0.06	P	20
560	M	J	control	+	29.32	+				0.06	F	9
594^e^	M	J	control	+	29.60	+	+			0.16	P/F^d^	14
636^e^	M	A	control	+	33.92	+		inc		2.62	F	15
690^e^	M	J	control	+	31.77	+				0.06	F	9
890	F	p	none	+	26.15		+			0.06	n/a	15
892	M	p	none	+	26.50	+				0.06	F	18
895	F	p	none	+	32.89	+	+			0.06	F	10
899	F	p	none	+	29.48	+	+			0.06	P	18
669	F	p	none	+	30.53		+			0.06	F	17
**VACCINATED**												
598^c^	M	A	topical^b^	–	n/d	–				9898.00	P^c^	50
599	M	J	topical	–	n/d	–				n/a	P	18
614	M	A	topical ^b^	–	n/d	–				0.06	P/F^d^	23
687^a^	M	A	oral	–	n/d	inc				0.40	F	12
638^a^^,^^e^	M	A	oral ^b^	inc	38.05	–				0.08	P	22
595^a^	M	A	topical	inc	37.27	–				0.06	P	15
691^a^^,^^e^	M	A	topical ^b^	inc	37.14	–				3.42	P	29
597	M	A	oral	+	31.74				–	390.80	P	11
639^e^	M	A	oral ^b^	+	31.68	–				0.11	P	18
586	M	A	topical	+	29.99	–				0.06	F	11

^a^ Indicates bats that were excluded from the Fisher´s exact test to compare the proportion of RABV shedding in saliva or salivary gland of bats by vaccination group

^b^ Bats that received a booster 100 days after initial vaccination

^c^ This bat showed progressive loss of movement and coordination, consistent with rabies but remained alive through the 50-day observation period. It was confirmed RABV positive. We tested nine of his oral swabs collected between the initial observation of clinical signs until euthanasia; the results were all negative.

^d^ Bats 594 and 614 initially displayed signs corresponding to the paralytic presentation (incoordination, unresponsiveness) but behaved furiously before death (self-inflicted wounds, aggressive behavior, and excitability).

^e^ Bats tested to confirm the cRABV of canine origin (coyote) using end-point RT-PCR and Sanger sequencing.

Preliminary Kaplan-Meier analysis provided no evidence of differences in survival proportions of bats sub-grouped by route (topical or oral) or boosting in vaccinated bats. Therefore, we combined bats that received one or two vaccine doses either topically or orally into vaccine treatment groups and compared their survival to controls (RCN-*luc*), by serostatus at baseline. The seven bats involved in the natural outbreak are included in their respective treatment groups, as removing them did not change the results. Survival of females (n = 21, excluding the only seropositive bat that received no treatment) was analyzed separately. Pups were considered a “RABV naïve” group.

**Fig 2:**
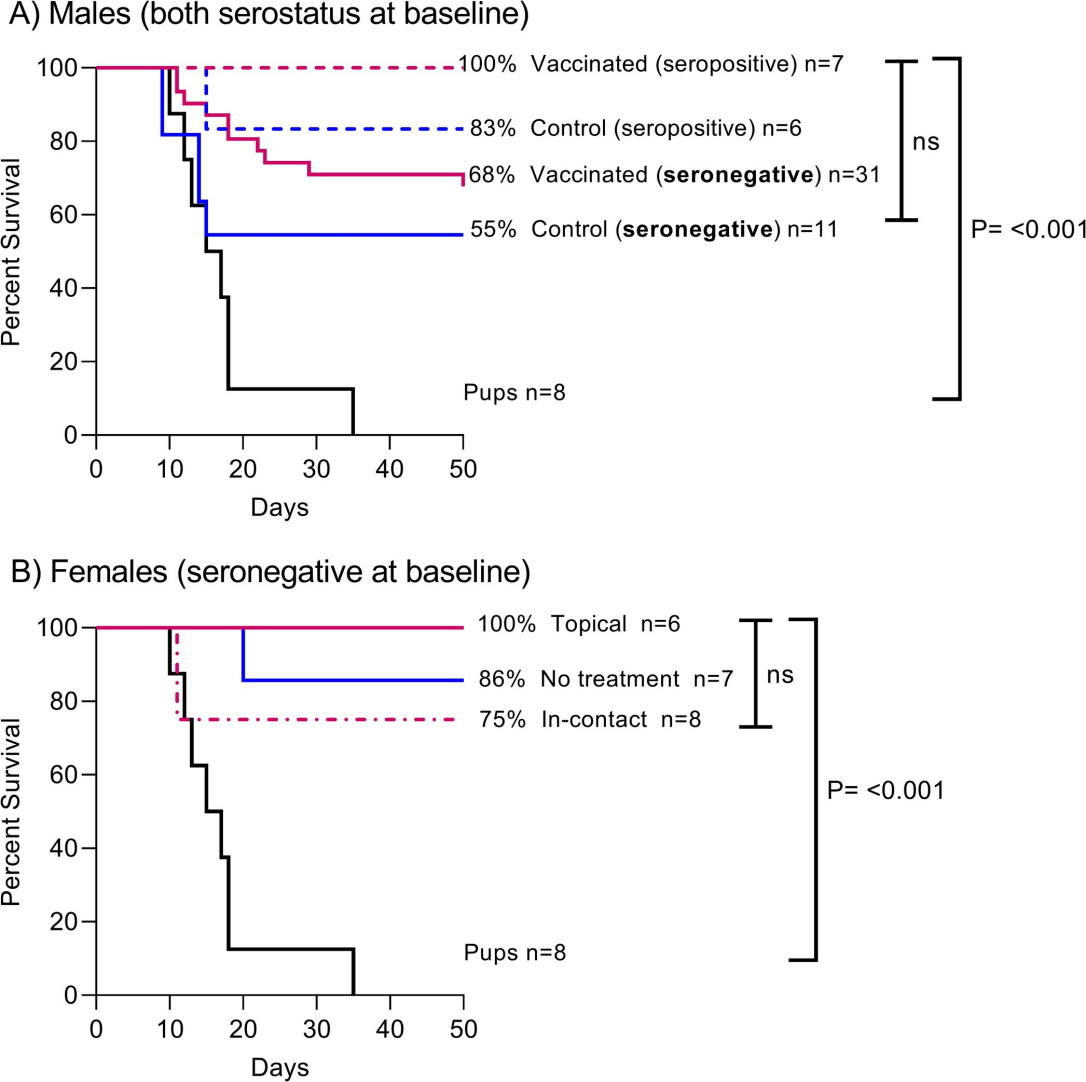
Kaplan-Meier analysis of vampire bat survival after challenge with a heterologous RABV strain and observation for 50 days post-challenge in A) male bats grouped by initial serostatus (seropositive/seronegative) and treatment received (vaccinated orally and topically, or control, RCN-*luc*), and B) female bats, seronegative-at-baseline and grouped by treatment received (vaccinated topically, in-contact, or no treatment). Eight captive-born pups were considered RABV naïve controls. A P value of < 0.05 was set as significant and “ns” indicates no significance. The numbers of animals in each group are indicated.

The overall survival proportions of males by treatment group were 100% (7/7) of seropositive-at-baseline vaccinated (orally) bats, 83% (5/6) of seropositive-at-baseline control bats, 68% (21/31) of seronegative-at-baseline vaccinated bats, 55% (6/11) of seronegative-at-baseline control bats, and 0% pups (no treatment). Females also survived in high proportions, with 100% (6/6) survival of topically vaccinated bats, 86% (6/7) of untreated bats, and 75% (6/8) of “in contact” females (housed with topically vaccinated females). Kaplan-Meier survival analyses showed no difference in survival among treatment groups for males and females. However, a significant difference in survival was detected when pups were included in the analyses for both sexes (P = <0.0001) (Fig 2). Also, no significant difference (P = 0.20) in survival was noted in vaccinated bats that seroconverted compared to those that did not.

### Survival in relation to serostatus

**Fig 3:**
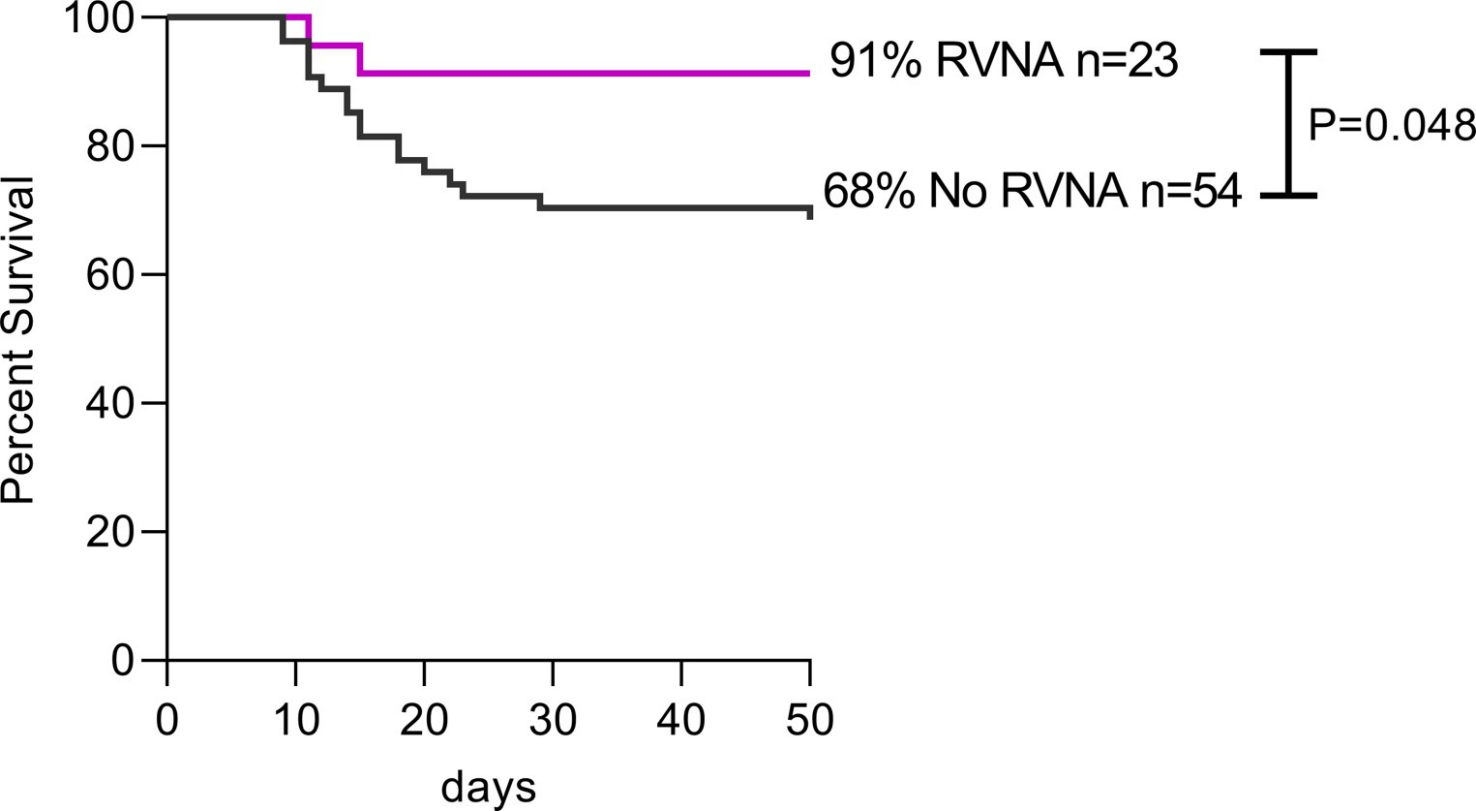
Kaplan-Meier analysis of vampire bat survival after challenge with a heterologous RABV strain and observation for 50 days post challenge in vampire bats with rabies virus neutralizing antibody (RVNA), either naturally acquired or after vaccination, compared to vampire bats without RVNA. A P value of < 0.05 was set as significant and “ns” indicates no significance. The numbers of animals in each group are indicated.

Survival of vampire bats (excluding pups) was significantly higher (P = 0.048) in individuals with detectable RVNA, whether acquired naturally or after vaccination, compared to those without (Fig 3). However, no significant difference (P = 0.17) was noted in comparing only bats that seroconverted after vaccination to those that did not. All but one of the 8 seronegative-at-baseline (87%), vaccinated males that seroconverted survived challenge, but so did 57% of 21 bats that did not seroconvert. Six of 11 control bats that received RCN-*luc* and had no detectable RVNA also survived RABV challenge. Nearly all seropositive-at-baseline males survived challenge (10/11), which included four seropositive bats that survived both the natural outbreak and cRABV challenge. Three other males that seroconverted after the outbreak or unexpectedly (one control) also survived challenge. Only one female had detectable RVNA, and she survived challenge along with 18/21 seronegative females. Finally, one male (#579) that survived both the natural outbreak and cRABV challenge and was orally vaccinated remained seronegative throughout.

Post-challenge, we detected RVNA in the majority (69%, 58/84) of the samples available at the end timepoint (i.e., death or end of study) collected up to 50 days after cRABV challenge. In contrast, 17 mature bats (nine unvaccinated and eight vaccinated) failed to show RVNA throughout the study, even after challenge. Yet, eight of these bats (three unvaccinated and five vaccinated, including the one associated with the natural outbreak) survived cRABV challenge and were rabies negative by DFA.

### Rabies virus shedding in saliva of infected vampire bats

To evaluate RABV shedding in saliva, we collected oral swabs periodically after challenge (e.g., each bat was swabbed every 3 days or daily if clinical signs were observed) and at time of death, if possible. Salivary glands were collected upon necropsy. We used RT-PCR to detect the presence of RABV nucleic acid for both sample types. In Table 3, we report results of RABV detection in saliva and salivary glands of bats that died from rabies (no RABV was detected in bats that survived). Oral and topically vaccinated bats are grouped in a “vaccinated” category, and control bats (RCN-*luc*) and those that did not receive any treatment (i.e., pups, no treatment, and in-contact females) are grouped as “non-vaccinated,” irrespective of serostatus at baseline. Of the 27 bats confirmed rabies positive by DFA, four had missing saliva samples, or the RT-PCR result was repeatedly deemed “inconclusive” and not included in subsequent analyses. Most bats in the vaccinated group underwent the paralytic form of rabies without visible clinical signs and died suddenly, so they were not swabbed daily. Hence, a single saliva test result is shown for this group (Table 3). Similarly, RT-PCR results on salivary glands from three bats were consistently “inconclusive” and not included. As a control, salivary glands from five bats confirmed negative by DFA at the end of the study (one control, one topically vaccinated, two orally vaccinated, and one control from the natural outbreak) were also tested by RT-PCR. Their results (not shown in table) were negative.

For saliva samples, RABV nucleic acid was detected in 71% (10/14) of non-vaccinated bats that died from rabies (samples from two bats unavailable and one inconclusive). In contrast, none of the vaccinated bats that succumbed to rabies (n = 9; one was inconclusive) had detectable RABV nucleic acid in any samples tested post challenge. The proportion of shedding between vaccinated and non-vaccinated bats is significantly different (P = 0.0016). Removing the pups from this analysis did not change the result; RABV was detected at a significantly higher rate (P = 0.005) in non-vaccinated adult bats (5/7) compared to vaccinated adults (0/9). For the salivary glands, we detected RABV nucleic acid in 94% of the non-vaccinated group (16/17). The one bat with the negative result (#608, female, in-contact) had a positive RT-PCR saliva sample (Ct value = 30.4). In contrast, we detected residual RABV nucleic acid in 43% of salivary glands from vaccinated bats (three of seven including samples with Ct values 30 or higher), and the proportion of RABV nucleic acid detection between vaccinated and non-vaccinated groups was significantly different (P = 0.014) for this tissue sample. Bat #598 (seronegative, topically vaccinated, and DFA positive) was repeatedly sampled after showing signs of rabies; however, RABV nucleic acid was not detected from a total of nine saliva samples from this bat or its salivary gland. We could not isolate RABV from saliva or salivary glands using RTCIT from any of the RT-PCR positive bats sampled, suggesting the detection of residual viral RNA only. The lack of viral isolation may have been due to low sample volume or dilution.

## Discussion

An ideal vaccine candidate against rabies in bats would not only reduce infection of susceptible individuals (e.g., by eliciting a protective immune response) but also stop the spread of RABV among populations, to other animals, and even to humans. We found that oro-topical vaccination of vampire bats with RCN-MoG is safe and capable of stimulating the development of RVNA after vaccination, though except for the pups, no significant difference in survival was noted between treatment groups, whether seropositive- or seronegative-at-baseline, vaccinated or unvaccinated. More importantly, however, we found that vaccination blocked RABV shedding in saliva (the main mode of transmission of RABV) in bats that succumbed to the disease.

Of 85 bats challenged with heterologous cRABV, only 27, from all treatment groups and including all pups, succumbed to infection and were confirmed to be rabid by DFA (Table 3). Notably, RABV nucleic acid was detected by RT-PCR in the saliva of 71% of unvaccinated bats that died of rabies but not in the saliva of any vaccinated bats that died of rabies. Likewise, detection of RABV nucleic acid in salivary glands was significantly higher in unvaccinated bats that died (94%) compared to vaccinated bats that died (43%), though viable RABV could not be isolated in cell culture from any of the salivary glands. It is possible that some residual, non-degraded viral RNA remained sequestered within this tissue. These results indicate that shedding of RABV was blocked in bats vaccinated up to 127 days before RABV challenge. These observations are consistent with our prior report of vampire bats vaccinated during a natural outbreak of RABV [23]. Of nine bats that died during the outbreak previously reported, 5 were unvaccinated, and RABV nucleic acid was detected in their saliva. In contrast, no RABV RNA was detected in four bats vaccinated within one week after natural exposure [23].

Other studies on RABV infection of vampire bats have reported discrepancies in the detection of RABV nucleic acid in the salivary gland and saliva, possibly due to timing of sample collection, short incubation period, and RVNA titers [27,40,41]. Rapid death after infection (evidenced by short incubation times) may not allow RABV to reach the salivary gland and be excreted in the saliva [27]; or sampling may not have coincided with viral excretion (e.g., during intermittent shedding) [42]. These are not likely explanations in our study, as we serially swabbed bats when they showed signs of illness and at the time of death, and mean incubation times (time to death) between vaccinated and non-vaccinated groups were not significantly different (t = 1.71, df = 25, P = 0.099). Instead, our results indicate the lack of RABV in saliva and salivary glands of vampire bats was strongly associated with vaccine treatment. However, the mechanism by which RCN-MoG may hinder or block RABV shedding is unclear and deserves further study. Recombinant poxvirus vectors have the potential to induce strong humoral and cellular immune responses in the mucosa [43,44]. It is possible that strong cellular responses (not detected by RFFIT) may prevent replication of RABV in the salivary glands, thus impeding the excretion of RABV into the saliva. Other virally vectored vaccines have also been shown to block pathogen transmission (e.g., chimpanzee adenovirus and modified vaccinia virus Ankara expressing malaria antigens [45] or baculovirus expressing MERS-CoV spike S1 protein [46].

Our study was confounded by the occurrence of a natural rabies outbreak of vampire bat origin [23] in our captive animals 3 months after capture. For our experimental challenge study, we used a heterologous RABV strain of canine (coyote) origin, which confirmed that mortality was due to challenge and not due to natural transmission of the vampire bat rabies variant. The lethality of this strain in vampire bats was unknown before the challenge. As observed by others [16,17,19], even using homologous strains (of vampire bat origin) in experimental challenges does not always cause mortality or reproducible observations. Additionally, the route, dose, and inoculation site of RABV may affect the consistency of disease progression and mortality rates in bats [47]. In this study, challenging the pups confirmed the lethality of the cRABV strain since all succumbed to rabies. We assume the age of the pups at the time of challenge corresponded to that of a developed immune system, as no significant differences in mean time-to-death (a proxy for incubation) or presentation of disease was evident between pups and adults.

Our study was also complicated by the unknown history of rabies exposure in our bats, which may have affected response to vaccination and survival to challenge, confounding our assessment of vaccine immunogenicity and efficacy. Unexpectedly, a high proportion of unvaccinated bats, seronegative for RVNA at baseline (55%), survived rabies challenge. It is possible that some or many of these bats were previously exposed to RABV, as several of our capture sites in México were located in VBR endemic areas, but if so, we failed to detect RVNA. Possibly, their RVNA titers had dropped to undetectable levels at the time of baseline sampling, or other effectors of the immune response not measured, such as T-cell mediated cellular responses, played a role in clearing infection.

Others have observed declines in RVNA in bats over time [28,48], and we did in our study as well (Fig 1). For example, most females (11/15) had detectable RVNA 33 days after challenge (0.52–69.9 IU/mL), but these titers declined significantly (0.12–4.82 IU/mL) by the end of the study (approximately 3 months later; Fig 1C). Waning of RVNA can be highly variable [17,48,49]. In this study, we observed some bats whose RVNA declined after 43, 91, or 117 days from a previously seropositive reading. Based on our highly variable observations, we speculate that the duration of detectable RVNA may not only vary as a function of amount, route, or timing of exposure to RABV, but also on how often an individual is re-exposed to RABV during its lifetime. Abortive infections occasionally occur in the wild [50], allowing individuals to resist rabies infection even after repeated exposure to the virus [28,48] and resulting in RVNA seroprevalence during screening. In our study, 14.7% (13/88) of bats exhibited RVNA at the beginning of the experiment. All but one of these ultimately survived challenge, with no differences noted between vaccinated and unvaccinated bats.

In male bats designated seronegative-at-baseline, vaccination with RCN-MoG elicited RVNA production in only 28% (8/29), seven orally and one topically vaccinated. Boosting did not improve seroconversion rates but seemed to elicit a more robust response, as the two bats (2/17) that seroconverted after the booster showed higher titers than those not boosted. In topically vaccinated bats, ingestion of the vaccine was corroborated by the presence of biomarker in hair samples. Still, dosages for topical application have not been optimized yet and may have been insufficient to elicit an antibody response. Others have observed highly variable seroconversion rates (e.g., 25–95%) in vampire bats after vaccination with recombinant vaccines using the oral or parenteral [16,18,19], and topical routes [19], and in another bat species, *E*. *fuscus*, using RCN-MoG topically (0%) and orally (22%) [15].

Survival of vampire bats upon cRABV challenge was higher (P = 0.048) in individuals with detectable RVNA, either acquired naturally or after vaccination, compared to bats with no detectable RVNA any time prior to challenge. Of 23 bats with detectable RVNA, 91% survived cRABV challenge, but 68% with no detectable antibody prior to challenge also survived. Moreover, eight bats that survived cRABV challenge failed to show RVNA even after challenge, and one of those had survived both the natural outbreak and the experimental challenge. As observed previously [15,16,18], survival after RABV challenge can occur in the absence of detectable neutralizing antibodies (elicited by vaccine or natural infection), indicating that alternative immune response mechanisms may operate against RABV in vampire bats (i.e., cell-mediated immunity or innate) [51,52]. Still, clear immune response pathways remain poorly understood in bats [10,53] and alternative methods to measure other effectors of the bat’s immune system, such as cell activation assays to detect cellular responses, could be considered in future vaccination trials.

Although vaccination of vampire bats in our study did not enhance survival after rabies challenge above that provided by natural or previously acquired immunity, our results are still encouraging in that vaccination apparently blocked RABV shedding in bats that succumbed to the disease. Thus, vaccination could potentially manage and control VBR without culling by preventing viral spread from rabid bats to other animals, not by increasing the survival of vampire bats. To confirm this finding and more thoroughly test the effects of vaccination, future trials would benefit from using established colonies of vampire bats that can be guaranteed “free” of rabies, e.g., born in captivity.

Even though rabies vaccination programs have significantly lowered the incidence of rabies in terrestrial wild animal populations [5] and could be useful in vampire bats, we recognize this is a controversial prospect and additional data on vaccine safety, especially for non-target species, effective delivery methods, and effects of vaccination on vampire bat populations are still needed. Because orally vaccinated vampire bats may carry the viral vector, RCN, in their mouth, they could potentially transmit the virus to cattle when they bite them. Other investigators have shown that oral ingestion of RCN-based vaccines is highly safe in numerous species [54,55]. Still, additional safety studies are needed to confirm transmission of the virus intradermally would not cause significant disease in cattle or other non-targets. Limited human infection with RCN was documented after an accidental needle stick in the laboratory [56]; a single pox lesion was evident at the inoculation site and did not spread. As for delivery of vaccine, topical application is the most feasible method for immunizing wild bats, either by hand or using spray devices. Due to high levels of allogrooming among vampire bats [57], transfer of vaccine would be highly likely, much like transfer of vampiricides used for culling bats. Studies to optimize the methods and amounts of vaccine to apply topically in various field settings are needed before field efficacy trials are attempted.

Finally, in addition to the risk of transmitting rabies, vampire bat predation on livestock, in the absence of rabies, can be a significant cause of economic losses in most Latin American countries [58]. Some believe that vaccinating bats to protect them against rabies will increase their abundance, but limited evidence exists to support this concern. A high proportion of vampire bats survive rabies naturally, as evidenced by high seroprevalence in wild populations, and modeling based on available data has estimated that vampire bats rarely develop lethal rabies infection (~10% of exposures) [59]. Therefore, it is unlikely that rabies acts as a form of population control. Other factors have been shown to be associated with vampire bat abundance, such as prey availability (primarily livestock) [60] or additional suitable habitat (e.g., man-made roosting sites). The consensus among farmers and other stakeholders is to eliminate vampire bats [61], even when rabies outbreaks do not occur, and culling vampire bat populations has been, for decades, the main practice to mitigate VBR. Thus, introducing vampire bat vaccination as an alternative strategy, even to prevent livestock losses, will require breaking longstanding paradigms. A vaccine that does not increase survival but helps reduce viral transmission may be very attractive. Even so, other strategies (non-lethal) aimed at controlling bat abundance would be needed, such as the use of contraceptives, which ideally could be delivered topically along with rabies vaccine.

The results of our study should encourage further investigation on rabies vaccination of vampire bats and motivate field trials with wild populations on a manageable-sized scale. In combination with other strategies aimed at reducing bat abundance and predation, this approach may show promise in reducing the burden of VBR in Latin America.

## Supporting information

S1 Fig Phylogenetic tree discriminating between vampire bats and coyote strains of rabies viruses. (PDF)
