# Supplementary material for: A recombinant rabies vaccine that prevents viral shedding in rabid common vampire bats (Desmodus rotundus)
Source: PLoS Negl Trop Dis. 2022 Aug 26;16(8):e0010699. doi: 10.1371/journal.pntd.0010699 (PMC9455887; doi:10.1371/journal.pntd.0010699)
Supplement: S1 Fig — (PDF) [file pntd.0010699.s001.pdf]

Supplemental information

A recombinant rabies vaccine that prevents viral shedding in rabid common vampire bats (*Desmodus rotundus*)

Running title: Rabies vaccination of vampire bats blocks viral shedding

Elsa M. Cárdenas-Canales [1], Andres Velasco-Villa [2], James A. Ellison [2], Panayampalli S. Satheshkumar [2], Jorge E. Osorio [1]*, Tonie E. Rocke [3]*

[1] Department of Pathobiological Sciences, School of Veterinary Medicine, University of Wisconsin-Madison, Madison, Wisconsin, United States of America

[2] Poxvirus and Rabies Branch, Division of High-Consequence Pathogens and Pathology, National Center for Emerging and Zoonotic Infectious Diseases, Centers for Disease Control and Prevention, Atlanta, Georgia, United States of America

[3] US Geological Survey, National Wildlife Health Center, Madison, Wisconsin, United States of America

* Corresponding authors

E-mail: trocke@usgs.gov (TER), jorge.osorio@wisc.edu (JEO)

Disclaimer

The findings and conclusions in this report are those of the authors and do not necessarily represent the views of the Centers for Disease Control and Prevention. Any use of trade, firm, or product names is for descriptive purposes only and does not imply endorsement by the U.S. Government.

To confirm that bats succumbed to the RABV variant used in the challenge (from coyote origin), we selected eight bats from the 27 that succumbed after challenge and were rabies positive by direct fluorescent antibody testing (DFA) [1] . Using a homogenate of their brain sample, we performed an end-point reverse transcriptase polymerase chain reaction (RT-PCR) assay as described previously [2,3]. We used primers N921 F (YGTGTTCAAYCTHATYCACTT, genome position 991-1011) and 304 R (TTGACGAAGATCTTGCTCAT, genome position 1514-1533) to target for the partial length of the Nucleoprotein gene. The PCR products were purified using ExoSAP-IT™ PCR Product Cleanup Reagent (Applied Biosystems, California, USA) following manufacturer's instructions. Purified PCR product was sent out for commercial Sanger sequencing at the Biotechnology Center of the University of Wisconsin-Madison, Madison, WI, USA. The obtained sequences for the partial N gene were aligned using BLAST [4] and Clustal X [5] and further edited using BioEdit software [6]. Identity percentages to a rabies lyssavirus isolated from a coyote (accession number KU963495.1) were calculated and recorded using BioEdit and MEGA 7 [7]. A phylogenetic reconstruction to appreciate differences between a coyote and our vampire bat sequences was conducted using a maximum likelihood (ML) approach with a 264 bp nucleotides long fragment coding for the last 88 amino acids in the nucleoprotein gen (Supplemental Figure 1). MEGA 7 software was run implementing the GTR + I + G substitution model and 1000 bootstrap iteration to estimate branch support values. Only robust bootstrap values (higher than 70) supporting specific host-associated viruses are depicted across the tree.

The ML tree shows the clear discrimination and phylogenetic distance between the challenge virus showed as highlighted purple sequences and the vampire bat virus, highlighted as red sequences, that was present during the natural rabies outbreak in the captive cohort of vampire bats.

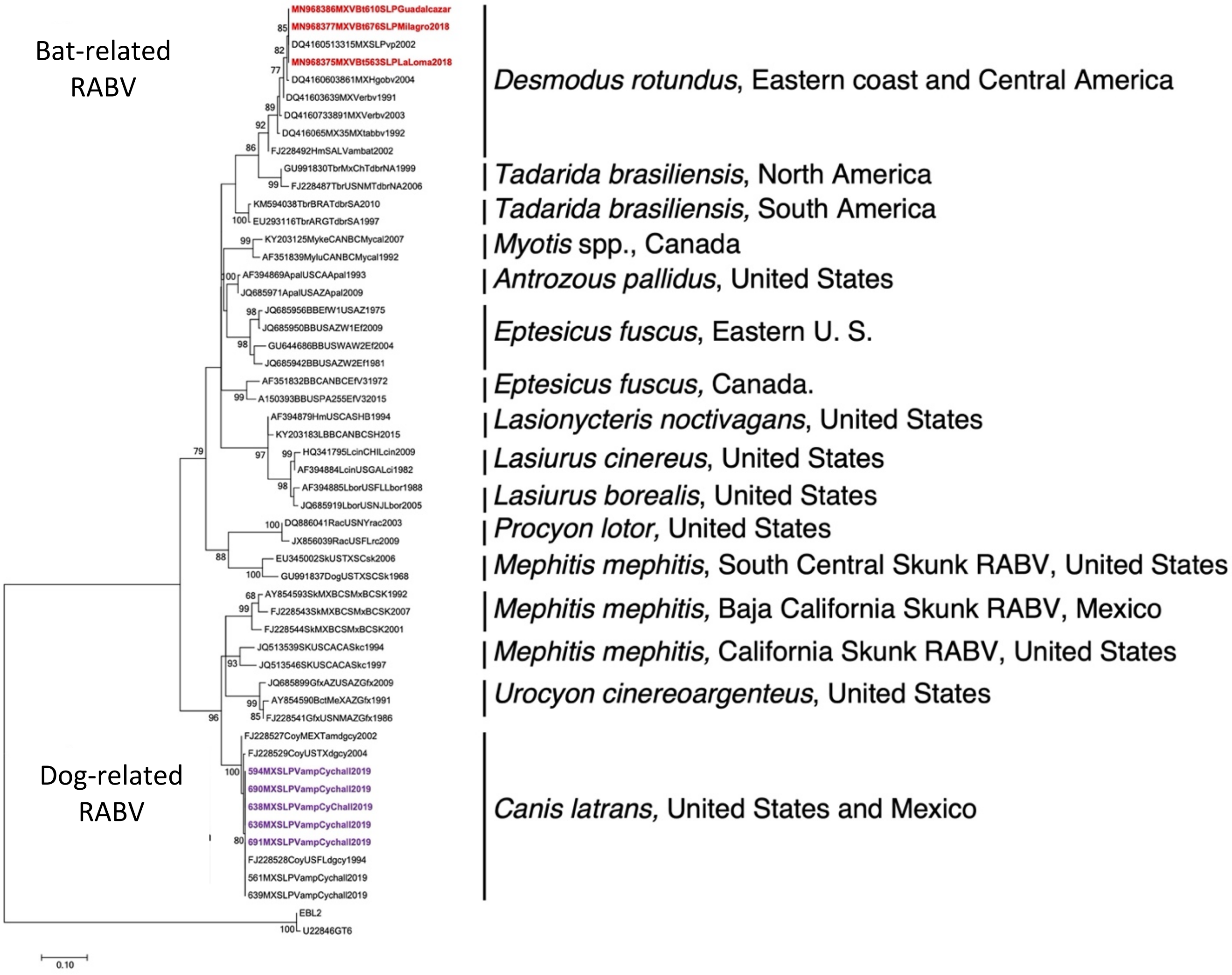

Figure S1. Phylogenetic tree discriminating between vampire bats and coyote strains of rabies viruses.

References

1. CDC. Protocol for Postmortem Diagnosis of Rabies in Animals by Direct Fluorescent Antibody Testing, A Minimum Standard for Rabies Diagnosis in the

United States. Available from: http://www.cdc.gov/ncidod/dvrd/rabies/Professional/publications/DFA_diagnosis/DFA_protocol-b.htm. 2006.

2. Binkley L, Deressa A, Shi M, Jara M, Escobar LE, Mauldin MR, et al. Use of partial N-gene sequences as a tool to monitor progress on rabies control and elimination efforts in Ethiopia. Acta Trop. 2021 Sep;221:106022.
3. Seetahal JFR, Velasco-Villa A, Allicock OM, Adesiyun AA, Bissessar J, Amour K, et al. Evolutionary History and Phylogeography of Rabies Viruses Associated with Outbreaks in Trinidad. Charrel R, editor. PLoS Negl Trop Dis. 2013 Aug 22;7(8):e2365.
4. Altschul SF, Gish W, Miller W, Myers EW, Lipman DJ. Basic local alignment search tool. J Mol Biol. 1990 Oct;215(3):403–10. AVailable from: https://blast.ncbi.nlm.nih.gov/Blast.cgi?PROGRAM=blastn&PAGE_TYPE=BlastSearch&LINK_LOC=blasthome. Accessed March 2020.
5. Larkin MA, Blackshields G, Brown NP, Chenna R, McGettigan PA, McWilliam H, et al. Clustal W and Clustal X version 2.0. Bioinformatics. 2007 Nov 1;23(21):2947–8. doi: 10.1093/bioinformatics/btm404.
6. Hall TA. BioEdit: A user-friendly biological sequence alignment editor and analysis program for Windows 95/98/NT. In: Nucl Acids Symp. 1999. p. Ser. 41: 95-98.
7. Kumar S, Stecher G, Tamura K. MEGA7: Molecular Evolutionary Genetics Analysis Version 7.0 for Bigger Datasets. Mol Biol Evol. 2016 Jul 1;33(7):1870–4.
